# The elephant trunk: a rare morphology of the left atrial appendage—a case report

**DOI:** 10.1093/ehjcr/ytae334

**Published:** 2024-07-18

**Authors:** Patrick Fischer, Felix Mahfoud, Michael Böhm, Christian Ukena

**Affiliations:** Klinik für Innere Medizin III, Kardiologie, Angiologie und Internistische Intensivmedizin, Universitätsklinikum des Saarlandes, Saarland University, Kirrberger Str. 100, Geb. 41.1, 66421 Homburg (Saar), Germany; Klinik für Innere Medizin III, Kardiologie, Angiologie und Internistische Intensivmedizin, Universitätsklinikum des Saarlandes, Saarland University, Kirrberger Str. 100, Geb. 41.1, 66421 Homburg (Saar), Germany; Klinik für Kardiologie, Universitätsspital Basel, Basel, Switzerland; Klinik für Innere Medizin III, Kardiologie, Angiologie und Internistische Intensivmedizin, Universitätsklinikum des Saarlandes, Saarland University, Kirrberger Str. 100, Geb. 41.1, 66421 Homburg (Saar), Germany; Klinik für Innere Medizin III, Kardiologie, Angiologie und Internistische Intensivmedizin, Universitätsklinikum des Saarlandes, Saarland University, Kirrberger Str. 100, Geb. 41.1, 66421 Homburg (Saar), Germany; Medizinische Klinik II, Kardiologie/Angiologie, Marien Hospital Herne, Universitätsklinikum der Ruhr-Universität Bochum, Herne, Germany

**Keywords:** Left atrial appendage, Interventional closure, LAAC, Atrial fibrillation, Anticoagulation, Case report

## Abstract

**Background:**

Patients with atrial fibrillation (AF) are at increased risk for thromboembolic events including stroke. The primary source for thromboembolism in these patients is thrombus formation in the left atrial appendage (LAA). Depending on the individual thromboembolic risk, long-term anticoagulation is recommended. In certain patients, however, long-term anticoagulation is contraindicated, and interventional closure of the LAA (LAAC) represents an alternative approach to lower the thromboembolic risk and avoid oral anticoagulation.

**Case summary:**

An 83-year-old male underwent LAAC at our centre in November 2022. Prior to the procedure, a thrombus in the left atrium (LA) or LAA was excluded by transoesophageal echocardiography (TOE), and the anatomy of the LAA was assessed as eligible for LAAC with no evidence of anatomical irregularities. After contrast medium injection, angiography revealed an atypical anatomic variant of the LAA with a substantially long, elephant trunk–like course.

**Discussion:**

We present a previously not described unique anatomic variant of the LAA: the elephant trunk morphology. Left atrial appendage anatomy is very heterogeneous, and detailed knowledge of LAA morphology is important for endovascular LAA procedures as well as for predicting the risk of thromboembolic events. Despite thorough pre-procedural imaging, anatomic variants may remain obscured.

**Learning points**

- In this case report, we present a previously undescribed atypical anatomic variant of the left atrial appendage (LAA): the elephant trunk morphology. It is distinguished by a substantially long and predominantly vertical course as well as a limited and constant diameter beyond the os.
- The LAA anatomy is very heterogeneous, and detailed knowledge of LAA morphology is important for endovascular LAA procedures as well as for predicting the risk of thromboembolic events.
- Despite thorough pre-procedural imaging, anatomic LAA variants may remain obscured.

## Introduction

Patients with atrial fibrillation (AF) are at increased risk for thromboembolic events including stroke. The primary source for thromboembolism in these patients is thrombus formation, which occurs in ∼90% of non-rheumatic AF in the left atrial appendage (LAA).^1^ Depending on the individual thromboembolic risk as assessed by the CHA2DS2-VASc score, long-term anticoagulation is recommended. In certain patients, however, long-term anticoagulation is contraindicated, e.g. due to prior severe bleeding events. In these cases, interventional closure of the LAA represents an alternative approach to lower the thromboembolic risk and avoid oral anticoagulation, which is also recommended by current guidelines.^2^ Interventional closure of the LAA (LAAC) is performed through femoral venous access and transseptal approach and guided by fluoroscopic and transoesophageal echocardiography (TOE). The LAA is a structurally very variable unit in which a variety of different shapes and orifice types are described. The LAA shape can increase the technical challenge of LAAC; therefore, pre-procedural planning and understanding LAA-specific anatomical characteristics are crucial for successful LAAC.^3^ In this case report, we present a previously not described atypical anatomical variant of the LAA, discovered unexpectedly during a LAAC procedure.

## Summary figure

**Figure:**
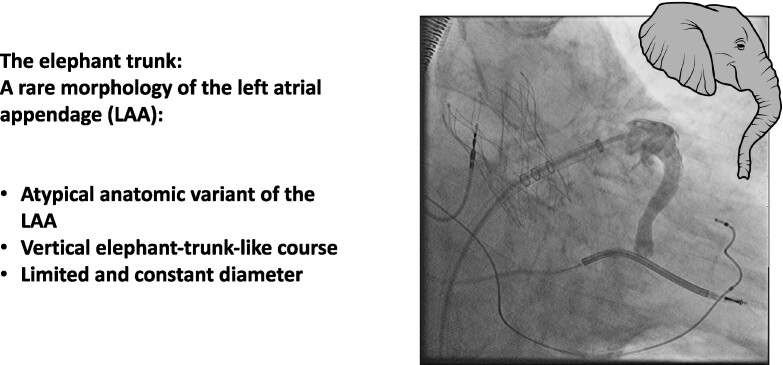


## Case report

We present the case of an 83-year-old male who underwent LAAC at our centre in November 2022. Past medical history included severe coronary artery disease with ischaemic cardiomyopathy and cardiac resynchronization therapy defibrillator (CRT-D) implantation in 2009. Due to high-grade aortic valve stenosis, transcatheter aortic valve implantation (TAVI) was performed in September 2022. The patient was also diagnosed with peripheral artery disease.

Due to paroxysmal AF and increased risk for thromboembolism (CHA2DS2-VASC score of 5), long-term anticoagulation with apixaban was prescribed. The patient received a dose of 2.5 mg of apixaban twice daily, meeting the criteria for dose reduction due to age over 80 years and creatinine levels >1.5 mg/dL. The patient experienced recurrent major gastrointestinal bleeding while on the reduced dose of apixaban, which resulted in consideration for LAAC.

**Figure 1:**
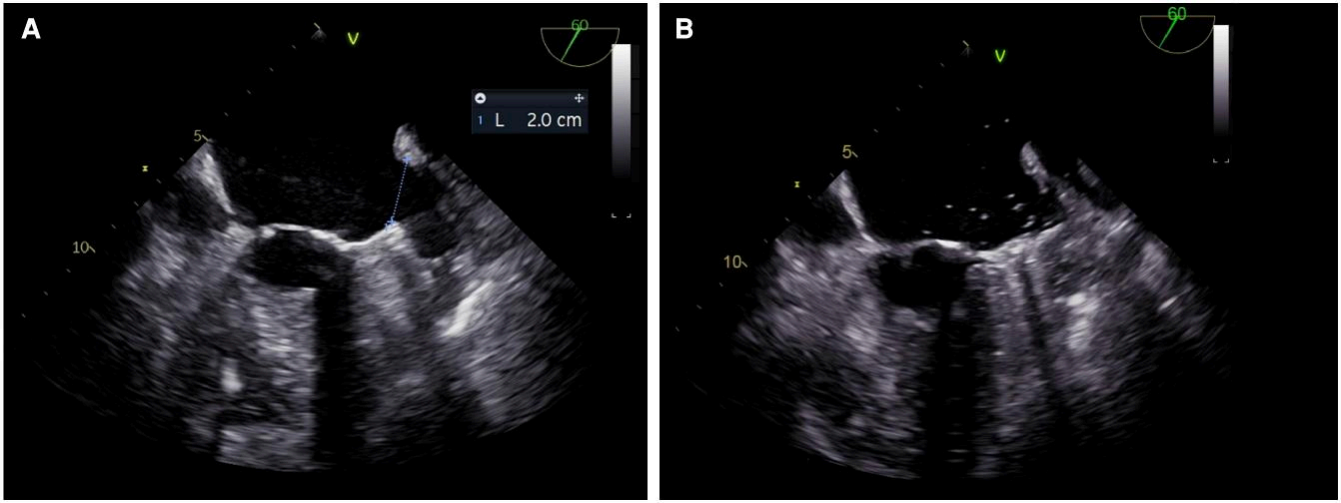
Pre-procedural (*A*) and post-procedural (*B*) 2D transoesophageal echocardiography. Left atrial appendage ostium measured 20 mm.

**Figure 2:**
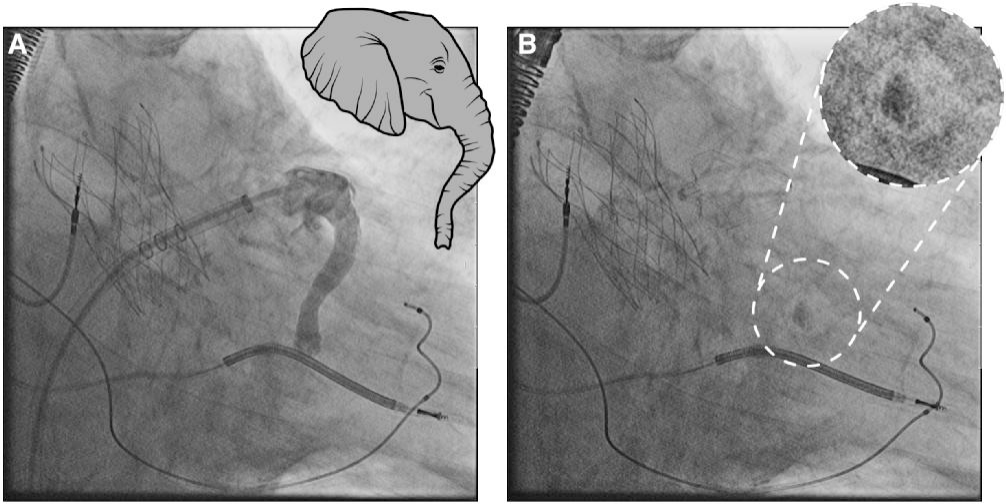
(*A*) Angiography of the left atrial appendage with a prolonged elephant trunk–like course. (*B*) Final result implantation of a Watchman 27 mm. Contrast medium remained in the tip of the left atrial appendage, indicating a low left atrial appendage velocity [both right anterior oblique view (25°) with cranial angulation (10°)].

Prior to the procedure, a thrombus in the left atrium (LA) or LAA was excluded by TOE. The anatomy of the LAA was assessed as eligible for LAAC with no evidence of anatomical irregularities (*Figure 1*). After right femoral venous puncture and transseptal puncture (BRK XS Series transseptal needle, 71 cm, St. Jude Medical), the LAA was probed. Contrast medium was injected, revealing an atypical anatomic variant of the LAA with a substantially long, elephant trunk–like course with an approximate length of 47 mm, a predominantly vertical course, and a limited and constant diameter beyond the os (see *Figure 2*; Supplementary material, *Video S1*). Angiography identified no evidence of thrombus formation in the LAA trunk. The LAA ostium was 20 mm (in fluoroscopy and echocardiography), allowing implantation of the LAA occluder (occluder, Watchman^™^, 27 mm, Boston Scientific; sheath, Watchman^™^ TruSeal^™^ Access System Double Curve, 12 F, Boston Scientific) without difficulties (see Supplementary material, *Videos S2 and S3*). As a sign of the significantly reduced blood flow in the LAA, a small amount of contrast medium was still detectable at the distal end of the LAA after completion of the implantation (*Figure 2*). The procedure was completed without any complications, and there was no evidence of residual leakage. Dual antiplatelet therapy was initiated using ASA 100 mg/day and clopidogrel 75 mg/day and followed by a single antiplatelet therapy with clopidogrel 75 mg/day. The patient passed away 3 months after the intervention due to severe pneumonia, preventing any follow-up examinations from being conducted.

## Discussion

The purpose of this case report is to highlight the diversity of LAA anatomy. Several publications on the wide anatomical variety of the LAA have been published. Especially, four types of LAA morphology are typically described either by TOE, cardiac computed tomography, or magnetic resonance imaging: chicken wing type, windsock type, cauliflower type, and cactus type.^4,5^ We present a previously not described unique anatomic variant of the LAA: the elephant trunk morphology, which was revealed angiographically.

Knowledge of the LAA anatomy is crucial for successful implantation of the device and to keep peri-procedural complication rates low. Transoesophageal echocardiography and cardiac computed tomography angiography (CCTA) are both suitable for pre-procedural imaging, focusing on ruling out LAA thrombus, characterizing LAA anatomy, and obtaining LAA measurement.

Additionally, CCTA can aid in selecting sheaths, determining transseptal location, and identifying optimal fluoroscopic angles during the procedure.^3^

Despite pre-procedural evaluation of the LAA using TOE, its actual shape was not fully recognized. This may have been detected with additional CCTA imaging. Possible challenges associated with anatomic variations further ostial may well cause difficulties such as poor anchoring or incomplete sealing.

New technological options are available to more accurately determine LAA morphology, which can facilitate accurate pre-procedural planning and successful implantation. For example, Ciobotaru *et al*. created a 3D model printing after an initial unsuccessful LAAC due to unusual LAA anatomy. The 3D model helped identify an alternative access route, enabling a successful LAAC procedure to be performed.^6^ Shimura *et al*. developed an LAAC procedural simulation model using virtual reality (VR) technology, which was based on pre-procedural computed tomography. The VR image offered multiple views, providing a thorough understanding of the patient-specific anatomy, and allowing operators to simulate a virtual LAAC procedure.^7^

In addition, LAA morphology is relevant for the risk of thromboembolic events. It has been shown that the risk for thromboembolic events is lower in chicken wing morphology compared with non–chicken wing morphologies.^8^ Most likely, this results from a smaller LAA orifice area and a higher LAA velocity in chicken wing morphology.^9^ Comparison of LAA velocity between the elephant trunk type and the other known morphologies remains to be assessed systematically. However, the persistence of contrast medium in the LAA after implantation suggests a low LAA velocity.

Another rare occurrence involving the LAA is its connection to the left ventricle (LV) via an accessory pathway (AP), which has been documented only a few times. In cases requiring surgical ablation, the LAA was found to be diffusely adherent to the overlying LV.^10^ In another instance, a case of LAA–LV connection with a giant LAA was reported.^11^ Giant LAAs are caused by congenital dysplasia of the atrial muscle or are secondary to mitral valve. They should be considered in challenging cases involving left-sided AP.^11^

## Conclusions

In this case report, we present a previously undescribed atypical anatomic variant of the LAA. While traditional imaging modalities like TOE may not fully capture atypical LAA morphologies, understanding these variations is crucial for selecting appropriate devices, minimizing complications, and optimizing long-term outcomes in LAAC procedures.

In our case, despite the unknown atypical morphology of the LAA, the LAAC procedure was successful without complications. Incorporating advanced imaging techniques such as cardiac CCTA and emerging technologies like 3D printing and virtual reality simulations can improve pre-procedural planning and enhance procedural outcomes in patients with complex LAA anatomy.

## Supplementary Material

ytae334_Supplementary_Data

## Data Availability

The data underlying this article will be shared on reasonable request to the corresponding author.

## Lead author biography

Patrick Fischer has been working as a cardiology resident at Saarland University Hospital in Homburg (Saar) since 2019, with a particular interest in invasive cardiac electrophysiology as well as in heart failure. After graduating from school, he first studied electrical engineering and information technology before completing his studies in human medicine at the Ruprecht Karls University of Heidelberg between 2012 and 2018.
